# Thrombin generation by activated factor VII on platelet activated by different agonists. Extending the cell-based model of hemostasis

**DOI:** 10.1186/1477-9560-4-5

**Published:** 2006-04-21

**Authors:** Raul Altman, Alejandra Silvia Scazziota, Maria de Lourdes Herrera, Claudio Gonzalez

**Affiliations:** 1 Centro de Trombosis de Buenos Aires, Buenos Aires, Argentina; 2 Department of Pharmacology, School of Medicine, University of Buenos Aires, Argentina

## Abstract

**Background:**

Platelet activation is crucial in normal hemostasis. Using a clotting system free of external tissue factor, we investigated whether activated Factor VII in combination with platelet agonists increased thrombin generation (TG) in vitro.

**Methods and results:**

TG was quantified by time parameters: lag time (LT) and time to peak (TTP), and by amount of TG: peak of TG (PTG) and area under thrombin formation curve after 35 minutes (AUC→_35min_) in plasma from 29 healthy volunteers using the calibrated automated thrombography (CAT) technique. TG parameters were measured at basal conditions and after platelet stimulation by sodium arachidonate (AA), ADP, and collagen (Col). In addition, the effects of recombinant activated FVII (rFVIIa) alone or combined with the other platelet agonists on TG parameters were investigated. We found that LT and TTP were significantly decreased (p < 0.05) and PTG and AUC→_35min _were significantly increased (p < 0.05) in platelet rich plasma activated with AA, ADP, Col, and rFVIIa compared to non-activated platelet rich plasma from normal subjects (p = 0.01). Furthermore platelet rich plasma activated by the combined effects of rFVIIa plus AA, ADP or Col had significantly reduced LT and TTP and increased AUC→_35min _(but not PTG) when compared to platelet rich plasma activated with agonists in the absence of rFVIIa.

**Conclusion:**

Platelets activated by AA, ADP, Col or rFVIIa triggered TG. This effect was increased by combining rFVIIa with other agonists. Our intrinsic coagulation system produced a burst in TG independent of external tissue factor activity an apparent hemostatic effect with little thrombotic capacity. Thus we suggest a modification in the cell-based model of hemostasis.

## Background

Tissue factor (TF) exposed after endothelial injury binds FVIIa to initiate thrombin generation (TG). In addition Factor VIIa on surface of platelets may cause TG in the absence of TF involvement [1,2]. In this process the transbilayer movement of phosphatidylserine through the flip-flop mechanism [3,4] likely provides the necessary procoagulant phospholipid surface. Thus platelets play a role in thrombin formation and thrombin is a potent platelet activator. Platelets that adhere to the exposed subendothelium [5] after endothelial injury, are influenced by a number of different agonists in addition to thrombin. One can readily imagine that such agonists not only influence platelet adherence and aggregation but also thrombin generation.

Indeed we showed previously that platelets stimulated by arachidonic acid (AA) significantly increased TG by the intrinsic clotting pathway independent of external TF [6]. In platelet rich plasma (PRP) from healthy subjects, ADP accelerated and enhanced TF induced TG via stimulation of P2Y12 receptors [7].

One controversial issue is whether TF is present in platelets and/or circulates in blood in the form of cell-derived microparticles [8,9]. Although platelets could not contains TF, they can generate thrombin through a TF independent mechanism [2]. Because TF expression by vascular cells induces intravascular thrombosis [9], in this study, we used an intrinsic coagulation system, lacking external TF. We examined whether platelet activation by well- known platelet activators (sodium arachidonate, ADP, or collagen (Col)) influences TG induced in PRP by recombinant activated FVII (rFVIIa), a pathway relevant to the hemostatic process.

## Materials and methods

### Subjects

Twenty nine healthy volunteers, 20 women and 9 men ages 22 to 72, without a history of thromboembolic, hemorrhagic, or other known disease consented to participate in the study. The volunteers agreed to refrain from taking any drug for 10 days prior to venapucture.

An additional group of 30 healthy volunteers with similar basal characteristics was studied using low platelet agonists concentrations.

An informed consent was obtained from all volunteers before joining the study. Only subjects with a normal prothrombin time and activated partial thromboplastin time were included in the study. To confirm the absence of any drug affecting platelet function, platelet aggregation in PRP was measured photometrically in a double-channel Lumi-Aggregometer (Chrono-log Corp., Havertown, PA,USA.). Any volunteers showing an abnormal platelet aggregation response to AA or ADP, was discarded from the study.

### Reagents

Fluorogenic thrombin substrate, Z-Gly-Gly-Arg-AMC, (Bachem, Switzerland) was dissolved in DMSO at a concentration of 100 mmol/l. A stock solution was prepared containing 100 mmol/l fluorogenic substrate, 1 mol/l calcium chloride and Fluo Buffer (20 mmol/l HEPES, pH 7.35 containing 60 mg/ml bovine serum albumin (Sigma, St Louis MO, USA)). For TG assays, a working solution containing 2.5 mmol/l fluorogenic substrate, 100 mmol/l CaCl_2 _and 2.5% (v/v) DMSO was used.

Platelet activation agents used in the TG assay were 1) arachidonic acid (AA), sodium salt (from Biopool Ventura, Ca, USA), 0.625 and 0.125 mmol/L; 2) ADP (Sigma, St. Louis, MO, USA), 2 μmol/L; 3) collagen (collagen reagent Horm, from Dipro, Austria) 0.5 μg/ml; and 4) recombinant activated Factor VII (Novo Seven, NovoNordisk, Bagsværd, Denmark) generous gift from NovoNordisk, Argentina.

#### Preparation of plasma

Venous blood was withdrawn from the antecubital vein without stasis and mixed with 0.11 mol/L sodium citrate (1:10 v/v). Platelet rich plasma (PRP) was obtained by centrifugation at 900 rpm for 10 minutes at room temperature and platelet poor plasma (PPP) was obtained by centrifuging PRP at 4000 rpm for 10 minutes. PRP was adjusted to a platelet count of 290,000/μL to 310,000/μL with autologous PPP. If contamination of PRP with erythrocytes or leukocytes was observed by light microscopy, a second centrifugation at 900 rpm for 5 minutes was conducted to minimize the number of these cells. Plastic syringes, tubes, and pipettes were used for all tests.

#### Thrombin generation study

TG was assayed by the calibrated automated thrombography (CAT) technique essentially as described by Hemker et al [10]. An intrinsically triggered coagulation system was used in which platelets of PRP were activated with AA, ADP, Col, alone or in combination with rFVIIa. PRP and agonists were incubated for 10 minutes before adding rFVIIa and conducting the assay. Samples were assayed in round bottom polypropylene microtiter plates (Greiner Labortechnik, Germany) using a microtiter plate fluorometer (Fluoroskan Ascent reader, Thermo Labsystems Helsinki, Finland). Tests were run within 88 ± 24 minutes of blood collection.

The assay system consisted in 80 μL of PRP and 20 μL of a solution that brought the test mixture to a final concentration of 0.625 mmol/L and 0.125 mmol/L AA, 2 μM ADP, 0.5 μg/ml, collagen or 5, 2.6, 0.5, or 0.25 μg/mL rFVIIa. The fluorogenic substrate working solution was used to start the reaction. Fluorescence was measured at 15 second intervals over a period of 50 minutes. Each sample was assayed simultaneously in quadruplicate.

Platelet activation is a very sensitive process and activation during blood withdrawn or PRP preparation could affect the final results. To exclude this bias, experiments in which the lag time of thrombin generation was less than 1 S.D. shorter than the mean, were discarded.

#### Definitions used

Lag-time (LT): the time in minutes from the start of the assay to the initial generation of thrombin (the moment at which 10 nM thrombin is formed).

Time to peak of thrombin generation (TTP): the time in minutes required to reach maximum thrombin generation (TG).

Peak of thrombin generation (PTG): the maximum thrombin concentration expressed in nmol/L.

Endogenous Thrombin Potential (ETP): area under the curve (AUC) expressed in nmol/L of thrombin [11]. ETP was calculated and corrected for α_2 _macroglobulin-thrombin complex activity using thrombinoscope software, Maastricht, Netherlands considering the start tail at 35 minutes (AUCo→_35min_.)

#### Statistical analysis

The nature of the quantitative variables distribution was assessed by the Shapiro-Wilk test. Quantitative variables are expressed as mean ± standard desviation (SD). Differences among groups of data were explored through repeated measures ANOVA or Friedman tests according to the distribution. The Student-Newman-Keuls test and its "by ranks" version were used in the post hoc analyses. p < 0.05 (two tailed) were considered as significant.

CSS/Statistica, v.4.3, 1993, Software (StatSoft, Tulsa, USA) was used for analyses.

## Results

### Time parameters (LT and TTP)

In normal PRP activated by a high or low AA concentrations, a significant decrease in LT to TG was observed (Table 1). Likewise activation by ADP or Col significantly decreased LT but to a lesser extent than AA (Table 1). In addition, significant decreases in TTP of TG were observed in normal PRP activated by different agonists (Table 2). Because LT is a part of TTP the observed decreases in TTP likely result from the agonists effects on LT.

**Table 1:** Activating effect of agonists on Lag Time.

Assay System N-PRP plus	Lag Time (minutes) mean ± SD						
	
	B	C	D	E	F	G	H
	Saline	rFVIIa 5 μg/mL	p value B vs C	rFVIIa 2.6 μg/mL	P value B vs E	rFVIIa 0.5 μg/mL	p value B vs G
1. Saline	17.1 ± 5.1	7.9 ± 2.1	<0.001	8.2 ± 2.4	<0.001	9.0 ± 1.9	<0.001
2. ADP 2 μM	11.5 ± 3.0	7.2 ± 2.0	<0.001	7.6 ± 1.6	<0.001	8.1 ± 2.0	<0.001
3. Col 0.5 μg/mL	11.9 ± 3.1	7.0 ± 2.8	<0.001	7.9 ± 2.8	<0.001	8.4 ± 2.9	<0.001
4. AA 0.625 mmol/L	8.4 ± 2.0	5.9 ± 1.2	<0.05	6.2 ± 1.3	<0.05	6.8 ± 1.3	<0.05
5. AA 0.125 mmol/L	9.5 ± 2.7	6.5 ± 1.5	<0.05	7.0 ± 1.8	<0.05	7.3 ± 1.7	<0.05

Parameters of TG in PRP from 29 normal volunteers. Effect of ADP, collagen (Col); sodium arachidonate (AA) and rFVIIa at different concentrations.

Column B:1 vs 2 to 5 p < 0.01 Column C: 1 vs 4 and 5 p < 0.01; Column E. 1 vs 4 and 5 p < 0.005 for both; Column G. 1 vs 4 and 5 p < 0.01 for both.

**Table 2:** Activating effect of agonists on Time to Peak

Assay System N-PRP plus	TTP (minutes) mean ± SD						
	
	B	C	D	E	F	G	H
	Saline	rFVIIa 5 μg/mL	p value B vs C	rFVIIa 2.6 μg/mL	P value B vs E	rFVIIa 0.5 μg/mL	P value B vs G
1. Saline	21.8 ± 3.9	14.3 ± 3.1	<0.001	15.1 ± 3.2	<0.001	16.5 ± 3.2	<0.001
2. ADP 2 μM	16.8 ± 3.5	13.4 ± 2.8	<0.01	13.8 ± 2.4	<0.01	13.9 ± 3.0	<0.01
3. Col 0.5 μg/mL	16,6 ± 3.4	12.5 ± 3.2	<0.05	13.5 ± 3.1	<0.01	13.3 ± 3.1	<0.01
4. AA 0.625 mmol/L	13.5 ± 2.8	12.0 ± 2.2	NS	12.1 ± 2.6	NS	12.2 ± 2.5	NS
5. AA 0.125 mmol/L	14.6 ± 3.5	12.7 ± 2.6	NS	13.1 ± 2.7	NS	12.9 ± 2.5	NS

Parameters of TG in PRP from 29 normal volunteers. Effect of ADP, collagen (Col); sodium arachidonate (AA) and rFVIIa at different concentrations.

Column B:1 vs 2 to 5 p < 0.001 for all. Column C 1 vs 4 p = 0.05

Column E: 1 vs 4, 5 p < 0.01 and <0.05; Column G: 1 vs 2 to 5 p < 0.01.

Different concentrations of rFVIIa produced a significant, dose-dependent decreases in LT (Table 1). The highest dose of rFVIIa was more efficient in decreasing LT than any of the other agonists' (Table 1). In combination with other agonists rFVIIa significantly decreased LT compared to each agonist alone (Table 1, columns D,F,H). In contrast to AA, which significantly decreased LT in the presence of rFVIIa, ADP and Col did not significantly augment the effect of rFVIIa alone (Table 1, column C, E, G). In combination with ADP or Col, but not AA, rFVIIa further decreased TTP compared to ADP or Col alone (Table 2).

#### Amount parameters (PTG and AUCo→_35min_)

As is shown in Tables 3 and 4, all of the tested activators increased both the peak of TG and the AUCo→_35min _to significantly higher levels than observed for the saline control. Because there is a natural upper limit to both PTG and AUCo→_35min _values, it makes sense that, in general, we did not observe a dose dependency for rFVIIa further significant increase when one potent activator was added to another (Tables 3 and 4). Only the lowest concentration of rFVIIa increased PTG of the other agonists with the exception of the lower amount of AA (Table 3). Although there was an apparent tendency for higher TG using the lower AA concentration than for the higher concentration, differences were not statistically significant.

**Table 3:** Activating effect of agonists on Peak of TG (PTG).

Assay System N-PRP plus	PTG (nmol/L) mean ± SD						
	
	B	C	D	E	F	G	H
	Saline	rFVIIa 5 μg/mL	p value B vs C	rFVIIa 2.6 μg/mL	P value B vs E	rFVIIa 0.5 μg/mL	P value B vs G
1. Saline	97 ± 36	160 ± 36	<0.001	151 ± 42	<0.001	189 ± 42	<0.001
2. ADP 2 μM	156 ± 50	178 ± 49	NS	167 ± 45	NS	214 ± 70	<0.01
3. Col 0.5 μg/mL	158 ± 60	180 ± 45	NS	174 ± 46	NS	215 ± 63	<0.01
4. AA 0.625 mmol/L	160 ± 43	164 ± 42	NS	172 ± 49	NS	204 ± 59	<0.01
5. AA 0.125 mmol/L	191 ± 52	193 ± 52	NS	184 ± 49	NS	222 ± 69	NS

Parameters of TG in PRP from 29 normal volunteers. Effect of ADP, collagen (Col); sodium arachidonate (AA) and rFVIIa at different concentrations.

Column B:1 vs 2 to 5 p < 0.001 for all Column C 1 vs 5 p < 0.01, Column E: 1 vs 5 p < 0.05

**Table 4:** Activating effect of agonists on Area under the curve (AUCo→ _35min _).

Assay System N-PRP plus	AUC (nmol/L) mean ± SD						
	
	B	C	D	E	F	G	H
	Saline	rFVIIa 5 μg/mL	p value B vs C	rFVIIa 2.6 μg/mL	P value B vs E	rFVIIa 0.5 μg/mL	P value B vs G
1. Saline	934 ± 431	1770 ± 411	<0.001	1661 ± 363	<0.001	1850 ± 327	<0.001
2. ADP 2 μM	1523 ± 364	1873 ± 366	<0.05	1773 ± 349	<0.05	2078 ± 437	<0.01
3. Col 0.5 μg/mL	1430 ± 470	1883 ± 433	<0.01	1766 ± 384	<0.05	2032 ± 502	<0.01
4. AA 0.625 mmol/L	1520 ± 373	1711 ± 410	NS	1726 ± 404	NS	1934 ± 408	<0.01
5. AA 0.125 mmol/L	1754 ± 374	1991 ± 372	NS	1872 ± 355	NS	2115 ± 403	<0.01

Parameters of TG in PRP from 29 normal volunteers. Effect of ADP, collagen (Col); sodium arachidonate (AA) and rFVIIa at different concentrations.

Column B:1 vs 2 to 5 p < 0.01 for all. Column G: 1 vs 5 p < 0.01

From these results it follows that a synergistic effect of activators can only be observed using suboptimal activator doses. Therefore the effects of combining low amounts of agonists were also tested (Table 5). We found that, at the concentrations tested, ADP or Col had no additive effect to that of AA alone. A low concentration of recombinant tissue factor (TF) was more effective in shortening TG time parameters but not amount parameters, than any agonist alone or in combination.

**Table 5:** Effect of pair of agonists compared with single agonist in thrombin generationparameters (n = 30) using low doses agonists and rTF. Final concentration of agonists: ADP 0.2 μM; sodium arachidonate (AA) 0.125 mmol/L; collagen (Col) 0.05 μg/mL, recombinant tissue factor (rTF) 2.5 pmol/L

	B	C	D	E
	Lag Time (min.) mean ± SD	Time to Peak (min.) mean ± SD	PTG (nmol/L) mean ± SD	AUC (nmol/L) mean ± SD
1 ADP	15.4 ± 4.0	23.2 ± 4.9	155 ± 41	1260 ± 474
2. AA +ADP	11.3 ± 2.5	17.3 ± 2.6	198 ± 45	1604 ± 188
3. Col	14.3 ± 3.3	21.6 ± 3.7	148 ± 44	1392 ± 264
4. AA+Col	11.8 ± 2.5	17.8 ± 3.3	187 ± 53	1551 ± 257
5. AA	12.9 ± 3.1	19.0 ± 3.4	179 ± 58	1488 ± 286
6. rTF	5.8 ± 1.8	12.9 ± 2.4	154 ± 50	1495 ± 337

Parameters of TG in PRP from 30 normal volunteers. PTG: Peak to thrombin generation. AUC: Area under the curve. AA, ADP or Col were compared with their combining effects. Also rTF, 2.5 pM was compared with the other agonists.

Lag Time: 1 vs 2 <0.001; 3 vs 4 <0.01; 2 vs 5 = NS; 4 vs 5 = NS 6 vs 1 to 5 <0.001

Time to Peak 1 vs 2 <0.001; 3 vs 4 <0.001; 2 vs 5 = NS; 4 vs 5 = NS 6 vs 1 to 5 <0.001

PTG 1 vs 2 <0.001; 3 vs 4 <0.01; 2 vs 5 =NS; 4 vs 5 =NS 6 vs 1 to 5 =NS

AUCo→_35min _1 vs 2 <0.001; 3 vs 4 =NS; 2 vs 5 =NS; 4 vs 5 =NS 6 vs 1 to 5 =NS

rTF appears as more effective than each agonist alone or their combination in shorten times parameters of TG, but effects are not different when amount parameters are considered.

#### Comparison of the intrinsic clotting system with rTF-mediated clotting

**Figure 1:**
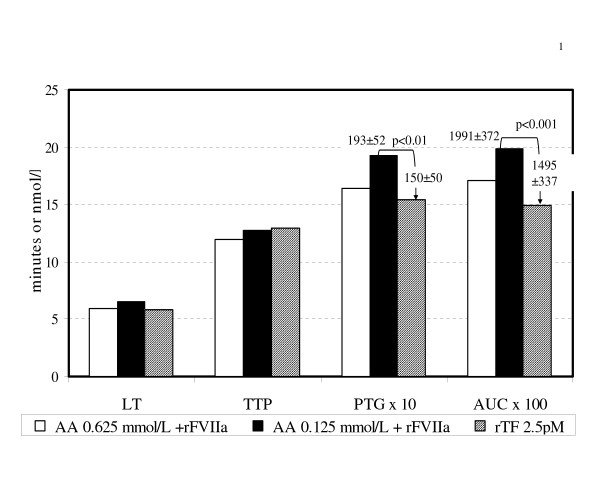
Effects of recombinant tissue factor (rTF) compared with sodium arachidonate (AA) at 2 concentrations (0.625 mmol/L and 0.125 mmol/L) plus recombinant activated FVII (rFVIIa) on thrombin generation parameters. Figure according to results of Table 1 to 4, lines 4 and 5, column C and Table 5, line 6, columns B to E. Concentration of rFVIIa: 5 μg/ml add after 10 minutes of PRP incubated with AA at 37^°^C. AA +rFVIIa time parameters (LT and TTP) are similar to those obtained by rTF in the indicated concentration. Although amount parameters were higher in the combined agonists compared with rTF, differences reach statistically significance when the lower dose of AA were employed (p < 0.01 and p < 0.001 for PTG and AUCo→_35min _respectively).

The amount of TF exposed after blood vessel wall injury is unknown, as is the contribution of soluble TF. However, it is probably much lower than the amount classically employed in tests of the Quick-time type. We arbitrarily used a rTF concentration of 2.5 pmol/L and compared its effects to the combined activity of AA at two concentrations plus rFVIIa. As shown in figure 1 the effects on TG were of the same order of magnitude.

## Discussion

Previously we found that AA-stimulation of platelets significantly increases TG in normal PRP by a pathway independent of external TF, shortening the LT and increasing the amount of thrombin formed [6]. Using the calibrated automated thrombography (CAT) technique [10] the present results confirms this finding and show that Col, ADP and rFVIIa also increase TG as measured by time (LT and TTP) and thrombin concentration (PTG and AUC) parameters. AA alone or combined with rFVIIa, was more effective in shortening time parameters than other agonists (Table 1 and 2) but all agonists similarly produced a small increase of amount parameters (Tables 3 and 4).

Because the physiological concentrations of ADP, Col and AA at the site of a lesion are unknown we hesitate to draw conclusions on the relative importance of these activators in vivo.

It must be taken into account that there is a natural upper limit to TG amount parameters and a natural lower limit to the time parameters. Therefore the potentiating effect of a second agonists is difficult to observe if the first agonist is already present in near optimal amounts. Nevertheless, potentiating effects were not observed even when low amounts of agonists were tested (Table 5).

We can speculate that the combined effect of AA and rFVIIa in the current system independent of external addition of TF resembles the effect of TF. Since the lower amount of AA plus rFVIIa was more effective in shortening the time parameters and in increasing the amount parameters than the higher amount of AA, it could be supposed that AA in combination with rFVIIa, has a concentration-dependent effect. (figure 1).

Because results were similar when ADP, Col, AA or rFVIIa were used at low agonists concentrations (Table 5), it is possible that the agonists and rFVIIa act through a common pathway. However other possibilities are also feasible: 1^st^. relative concentrations of activators and platelet receptors are important. 2^nd^. there is competition for platelets receptors between ADP/AA or Col/AA. Moreover there is not synergism between rFVIIa and the other activators since increased rFVIIa concentration alone or together with a fixed concentration of another agonist produced rFVIIa dose dependent activation.

Lisman et al [12] proposed that platelets are activated by collagen resulting in the exposure of procoagulant phospholipid surface. High-dose rFVIIa subsequently binds to the activated platelet, culminating in thrombin generation. In addition to Col, we found that AA and ADP are capable of activating platelets. Furthermore platelet activation could be accomplished by rFVIIa alone or in combination with another agonist.

Thus, the results obtained in the present in vitro studies, suggest that a burst in TG mediated by FVIIa plus other agonists independent of external TF may be important in normal hemostasis.

Whether this TG is independent of possible TF encrypted (not procoagulant) in platelets is beyond the scope of the current experiments. After undergoing the flip-flop mechanism, phosphatidylserine could cause decryption of platelet TF and may contribute to local hemostasis, but the potential importance of this activity is not well defined [13]

The burst of thrombin generated on the platelet surface produces a stable hemostatic clot [14]. As described by Hemker and Béguin [15] clotting begins when 10–20 nmol/L of thrombin is formed. Even if the initial amount of thrombin is not affected, the faster initial thrombin generation caused by agonists could be safe for patients and important for hemostasis. Clinical experience has shown a beneficial effect of rFVIIa in controlling bleeding in patients with various coagulation deficiencies or platelet defects [16]. It can be supposed that intravenously administrated rFVIIa will not act on surfaces devoid of adhered platelets resulting in enhanced of thrombin generation only at the site of vascular damage.

Considering our results and others from the literature [17-19] we can speculate that hemostasis and thrombosis mechanisms although similar proceed through different routes: rFVIIa can function via a TF-dependent or independent pathway and both pathways are operative in vivo [20]. Thus, although FVIIa activity is enhanced several-fold by endothelial exposed TF, external TF is not an absolute requirement since FVIIa can also generate thrombin on the platelet surface independently of external TF [1,2].

**Figure 2:**
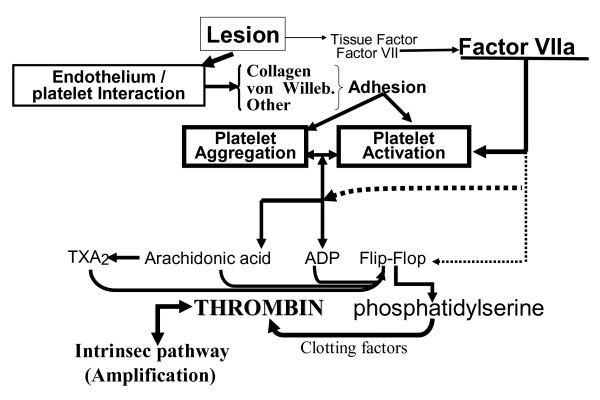
Extending the cell-based model of hemostasis. Hemostasis is the specific response to external vessel lesion and dependent on the extent of vessel wall damage; the specific interaction between endothelial cells and activated platelets; release of the contents of platelets intracellular granules in response to activation, the conjointly activity of activated Factor VII and platelet agonists; and the "open conditions" of blood flow.

In hemostasis, a small amount of exposed TF will transform FVII into it active form. With the help of agonists released in situ following platelet deposition on the wounded endothelium, FVIIa activates platelets and induces the membrane flip-flop mechanism. This leads to thrombin generation (first explosion) and local fibrin formation. The local clotting mechanism is limited by several factors: 1) dilution of activators in the bloodstream of a non occluded vessel; 2) a small amount of exposed TF in a relatively minor area of the wall lesion; 3) local inhibition of TF/FVIIa activity by tissue factor pathway inhibitor. 4) Limitation of clot extension by plasmatic inhibitors and 5) Limitation of clot lysis by platelet plasminogen activator inhibitor. Our model of hemostasis is in line with the cell-based model of coagulation [2,19] but the effects of agonists and their combined activities with FVIIa are included because they seem to be important in normal hemostasis (figure 2). In arterial thrombosis, mainly located on atheroma, inflammation promotes plaque rupture [21,22] and increases TF expression in endothelial cells and monocytes. The amount of TF bound to FVII (TF/FVIIa complex) is several times greater [15,23] than that of TF/FVIIa complex formed in a wounded vascular wall, and with platelet it causes a burst of thrombin formation (second explosion). Partial or total occlusion of the blood vessel, stasis and activation of blood clotting proteins and platelets and artery diameter, are parameters that prevent dilution of activated factors and generated thrombin. These parameters together with platelet-erythrocyte interactions [24] promote thrombus growth. Increased functional TAFI levels, resulting in decreased fibrinolysis, induce a more stable thrombotic plug and are associated with the risk of arterial thrombosis [25].

A practical point related to antithrombotic therapies arises. Increased concentrations of old or localized effects of new antithrombotic drugs will affect thrombosis as well as hemostasis but, because the latter is a weaker process, any increase in anticoagulant potential will produces a bleeding tendency before inhibiting thrombosis [26].

In conclusion, platelets activated by ADP, collagen or AA triggered TG in PRP. This effect was increased by the addition of rFVIIa. Although results of the current study cannot be directly extrapolated to patients, our findings allow speculation on the roles of platelet agonists and activated FVIIa in hemostasis and could provide new insights into antithrombotic therapies [27].

## Competing interests

One of the authors (CG) is Medical Director at Novo Nordisk Argentina, whose product NovoSeven was employed in this study. The interpretation of the results of this study are CG's personal opinion and do not in any way reflect those of Novo Nordisk.

## Authors' contributions

RA: conceived and had the main responsibility to designed the study and the conclusions, discuss the results, and drafted the manuscript. ASS: performed the clotting test, discuss the results and the conclusions and contributed to the drafting of the manuscript. MLH: performed the clotting test, discuss the results and contributed to the drafting of the manuscript. CG: Performed the statistical analysis, discuss the results, and contributed to the drafting of the manuscript.
